# Trajectory modeling of gestational weight: A functional principal component analysis approach

**DOI:** 10.1371/journal.pone.0186761

**Published:** 2017-10-24

**Authors:** Menglu Che, Linglong Kong, Rhonda C. Bell, Yan Yuan

**Affiliations:** 1 Department of Statistics and Actuarial Science, University of Waterloo, Waterloo, Ontario, Canada; 2 Department of Mathematical and Statistical Sciences, University of Alberta, Edmonton, Alberta, Canada; 3 Departement of Agricultural, Food and Nutritional Science, University of Alberta, Edmonton, Alberta, Canada; 4 School of Public Health, University of Alberta, Edmonton, Alberta, Canada; Universidade de Sao Paulo, BRAZIL

## Abstract

Suboptimal gestational weight gain (GWG), which is linked to increased risk of adverse outcomes for a pregnant woman and her infant, is prevalent. In the study of a large cohort of Canadian pregnant women, our goals are to estimate the individual weight growth trajectory using sparsely collected bodyweight data, and to identify the factors affecting the weight change during pregnancy, such as prepregnancy body mass index (BMI), dietary intakes and physical activity. The first goal was achieved through functional principal component analysis (FPCA) by conditional expectation. For the second goal, we used linear regression with the total weight gain as the response variable. The trajectory modeling through FPCA had a significantly smaller root mean square error (RMSE) and improved adaptability than the classic nonlinear mixed-effect models, demonstrating a novel tool that can be used to facilitate real time monitoring and interventions of GWG. Our regression analysis showed that prepregnancy BMI had a high predictive value for the weight changes during pregnancy, which agrees with the published weight gain guideline.

## Introduction

Normal physiological adaptations to pregnancy favor weight gain to support fetal growth, followed by weight loss postpartum to meet increased maternal energy demands during lactation [1, 2]. It is well known that the risk of poor maternal and fetal health outcomes increases when women gain either too little or too much weight during pregnancy. Gaining too little weight is associated with low birth weight and preterm birth [3]. Excessive weight gain in pregnancy, which is a growing problem in developed countries, contributes to increased rates of maternal and perinatal complications, including gestational diabetes, emergency caesarian delivery, large infant (>90% percentile) for gestational age at birth and sometimes even fetal death [4]. It has been reported that across all prepregnancy BMI categories, 45-80% of Canadian women gain in excess of their respective BMI category-specific Gestational Weight Gain (GWG) recommendations from Institute of Medicine [5–7]. Excessive GWG adds to the growing prevalence of overweight and obesity among women entering pregnancy (in the case of a subsequent pregnancy) and increases women’s long-term risk of obesity and obesity-related chronic diseases, such as diabetes, hypertension, and certain cancers [8].

Customized counseling and education combined with individual weight monitoring have been shown to improve GWG adherence [9, 10]. Women are currently referred to general weight gain trajectory based on their prepregnancy BMI category, but that adherence rates might be improved if weight trajectory could be individualized. Thus, monitoring and modelling individual weight growth trajectories during pregnancy could be clinically relevant and useful for women and health care providers. For example, the weight growth trajectory may be used to predict the gestational weight changes so that women at high risk of suboptimal GWG can be identified and appropriate intervention can be offered.

Instead of an individual’s unique trajectory, previous modeling studies on gestation weight growth aim to obtain a reference weight trajectory for the population of pregnant women [11–13]. Most statistical methods of modeling individual trajectory were developed for modeling the daily or monthly cycling patterns of certain biomarkers [14–16]. These mixed effects models require a large number of measurements per individual. This data requirement needs intensive data collection, which is challenging for large cohort studies over a 9-month pregnancy in the research setting. To reduce the data collection burden for both study participants and the research team, a model that can estimate individual trajectory using irregularly spaced sparse longitudinal data is needed. The search of suitable statistical method leads us to examine the functional principal component analysis (FPCA) approach [17]. The development of FPCA is motivated by irregularly spaced sparse functional or longitudinal data, where the functional principal component (FPC) scores are framed as conditional expectations. With a cohort of sparse longitudinal samples, FPCA is capable of efficiently estimating the individual continuous trajectory by borrowing information from the whole cohort.

We have three objectives in this study. First, to investigate whether the FPCA approach provides a good fit to individual gestational weight growth trajectory. Second, to compare the performance of FPCA to the classic method for trajectory analysis, i.e., the mixed effects model. Lastly, to examine the contribution of prepregnancy BMI to the variation in gestational weight changes.

## Materials and methods

### Materials

#### Data collection

In the Alberta Pregnancy Outcome and Nutrition (APrON) study, pregnant women who were less than 27 weeks of gestation were recruited. The study protocol has been published elsewhere [18]. The ethical approval is granted through University of Alberta Ethic Board (Human ethics PRO00002954). Briefly, upon a woman’s recruitment, informed written consent was obtained and her prepregnancy weight (*W*_0_) and due date were self-reported. Women recruited before 13 weeks gestation were assessed in each trimester, labeled as gestation stage A, B, C. Those recruited in 14-27 weeks gestation were assessed in gestation B and C. Each assessment included a weight measurement; a 24-hour food recall, either web-based to be completed online or interviewer-administered to be completed at a study site; a self-administered Baecke’s physical activity (PA) questionnaire, among other questionnaires. The web-based 24-hour food recall was developed by Hanning et al. [19] and the database was modified to include food items typically only consumed by adults, including alcohol [20]; and the interviewer-administered instrument was described by Conway et al. [21]. The Baecke’s PA questionnaire, first proposed by Baecke et al. [22], was modified for pregnancy [23]. In addition, height was measured at her first study visit, which was used to calculate her prepregnancy BMI. Gestational age (GA) was calculated based on due dates. Each women was asked to attend a postpartum visit at three months after delivery, during which her highest weight during pregnancy (*W*_*H*_) and delivery date were reported.

The weights measured in gestation stage A, B, C are denoted as *W*_*A*_, *W*_*B*_, and *W*_*C*_ respectively. In the 24-hour food recall, macro- and micro-nutrient intakes were calculated. A calibration study showed that direct pooling of the results from the web-based and interviewer-administered instrument may not be feasible [24] and therefore we model them separately. Levels of PA measured in the Baecke’s questionnaire were represented by the PA indices. Each subject had a maximum of 5 body weight data points, the self-reported *W*_0_ and *W*_*H*_ and the measured *W*_*A*_, *W*_*B*_, *W*_*C*_ for a trajectory, where corresponding GAs vary from 0 to 42 weeks. One or more of these data points could be missing due to missed study visit(s). We associate *W*_0_ to *t* = 0, and *W*_*H*_ to the GA at birth.

#### Inclusion criteria

Subjects who had a full-term (GA at birth ≥ 37 weeks), singleton live birth were included. For statistical modeling, we required the subject to have at least one record of physical activities or food intake, height measurement, *W*_0_ and at least one measured weight (*W*_*A*_, *W*_*B*_ and *W*_*C*_) with corresponding GA. Consistency is also required, for example, the GA at birth should be greater than the GA of *W*_*C*_.

### Method

#### FPCA set up and trajectory estimation

The Functional Principle Component Analysis (FPCA) approach proposed by Yao et al. [17] is developed for sparse, irregularly measured longitudinal data. We assume that each subject’s measurements come from a smooth underlying trajectory subject to measurement errors. The FPCA procedure first estimates the smooth covariance surface of the observed data as a function of time, then captures the principal components (PC) as functions of time that explain variance among all observed data. An estimated trajectory for each subject is reconstructed through a linear combination of the first few PC functions that captures most of the variation in the data. Since the PC functions are estimated from all available data, the reconstruction of trajectories borrows information from the entire sample. The validity of the FPCA approach is guaranteed even when there are only a few measurements for each subject. A mathematical overview of FPCA is provided below; readers who are less sophisticated in statistics may skip this section.

We consider a smooth random function *X*(*t*), with unknown mean *μ*(*t*) and smooth covariance function cov(*X*(*s*), *X*(*t*)) = *G*(*s*, *t*). Suppose that *X*_*i*_(*t*), *i* = 1, …, *n*, are its *n* realizations, each with *N*_*i*_ observations, *X*_*i*_(*T*_*ij*_) + *ϵ*_*ij*_, which were made at different time points *T*_*ij*_, *j* = 1, …, *N*_*i*_ and subject to uncorrelated measurement errors *ϵ*_*ij*_ with mean 0 and constant variance *σ*^2^. In our case, *X*(*t*) can be one of the trajectory of weight, nutrient intake, or PA index. The time *t* is in a closed time interval T between 0 and 42 weeks. In FPCA, the *j*–th observation of the *i*-th realization is expressed as

$$\begin{array}{c} Y_{ij}=X_{i}\left(T_{ij}\right)+\epsilon_{ij}=\mu \left(T_{ij}\right)+\sum_{k=1}^{\infty }\xi_{ik}\phi_{k}\left(T_{ij}\right)+\epsilon_{ij}, \end{array}$$ (1)

where *ϕ*_*k*_’s are the *k*-th principal component (PC) functions, assumed to be smooth, and *ξ*_*ik*_’s are the FPC scores that are uncorrelated with zero mean and unit standard deviation.

The estimation of the trajectory is implemented in the following four steps.

**Step 1** *Estimation of the mean function*.
  The mean function *μ* is estimated based on all the data points from all individuals, where local linear kernel smoothers proposed by Fan et al [25] are employed.
**Step 2** *Estimation of the covariance structure*.
  Let $G_{i}\left(T_{ij},T_{il}\right)=\left(Y_{ij}-\hat{\mu }\left(T_{ij}\right)\right)\left(Y_{il}-\hat{\mu }\left(T_{il}\right)\right)$ be the “raw” covariances of the observed data. Then we have
  $$\begin{array}{c} E\left[G_{i}\left(T_{ij},T_{il}\right)|T_{ij},T_{il}\right]\approx \text{cov}\left(X\left(T_{ij}\right),X\left(T_{il}\right)\right)+\sigma^{2}\delta_{jl}, \end{array}$$
  where *δ*_*jl*_ = 0 for *j* ≠ *l* and *δ*_*jl*_ = 1 for *j* = *l*. Therefore, we use only those *G*_*i*_(*T*_*ij*_, *T*_*il*_) with *j* ≠ *l* to first obtain the smoothed covariance surface of *X*(*t*) by the local linear kernel smoother, denoted by $\hat{G}\left(s,t\right)$. As noted by Yao et al [17], when the assembled pairs (*T*_*ij*_, *T*_*il*_) are sufficiently dense in the domain, the estimation of the covariance function is feasible. Next a local linear component along the diagonal and a quadratic component along the direction perpendicular to the diagonal are fitted. We denote the diagonal of the estimated surface by $\tilde{G}\left(t\right)$. A local linear kernel smoother $\hat{V}\left(t\right)$ focusing on diagonal entries of {*G*(*t*, *t*) + *σ*^2^} is obtained by using *G*(*T*_*ij*_, *T*_*ij*_) as input.
**Step 3** *Estimation of eigendecomposition*.
  The estimation of eigendecomposition, including the eigenfunctions (PCs) and eigenvalues, is essentially to solve the eigenequations
  $$\begin{array}{c} \int_{T}\hat{G}\left(s,t\right){\hat{\phi }}_{k}\left(s\right)ds={\hat{\lambda }}_{k}{\hat{\phi }}_{k}\left(t\right) \end{array}$$ (2)
  with respect to ${\hat{\phi }}_{k}$’s and ${\hat{\lambda }}_{k}$’s. These eigenfunctions are estimated by discretizing the smoothed covariance as in [26].
**Step 4** *Prediction of FPC scores*.
  To predict the FPC scores, the integration of a smooth function is needed, and general Riemann sums may not work well due to the sparse data. Denote ${\tilde{Y}}_{i}=\left(Y_{i1},...,Y_{iN_{i}}\right)$, then in FPCA through conditional expectation [17], the best prediction of the FPC scores is the conditional expectation $E(\xi_{ik}|{\tilde{Y}}_{i})$. If we further assume that *ξ*_*ik*_ and *ϵ*_*ij*_ are jointly normal, the conditional expectation is given by [27]
  $$\begin{array}{c} {\tilde{\xi }}_{ik}=E\left(\xi_{ik}|{\tilde{Y}}_{i}\right)=\lambda_{k}\phi_{ik}^{T}\Sigma_{Y_{i}}^{-1}\left({\tilde{Y}}_{i}-\mu_{i}\right), \end{array}$$ (3)
  where *ϕ*_*ik*_ = (*ϕ*_*k*_(*T*_*i*1_), …, *ϕ*_*k*_(*T*_*iN*_*i*__)), *μ*_*i*_ = (*μ*(*T*_*i*1_), …, *μ*(*T*_*iN*_*i*__)), and $\Sigma_{Y_{i}}=\text{cov}\left({\tilde{Y}}_{i},{\tilde{Y}}_{i}\right)=\text{cov}\left({\tilde{X}}_{i},{\tilde{X}}_{i}\right)+\sigma^{2}I_{N_{i}}$, is a *N*_*i*_ × *N*_*i*_ matrix, the (*j*, *l*) entry of which is *G*(*T*_*ij*_, *T*_*il*_) + *σ*^2^ *δ*_*jl*_, with *δ*_*jl*_ = 0 for *i* ≠ *j* and *δ*_*jl*_ = 1 for *i* = *j*. Plugging in the unknown quantities in Eq (3) by their estimates, we obtain the “plugging in” estimator of the FPC score
  $$\begin{array}{c} {\hat{\xi }}_{ik}=\hat{E}\left(\xi_{ik}|{\tilde{Y}}_{i}\right)={\hat{\lambda }}_{k}{\hat{\phi }}_{ik}^{T}{\hat{\Sigma }}_{Y_{i}}^{-1}\left({\tilde{Y}}_{i}-{\hat{\mu }}_{i}\right), \end{array}$$ (4)
  where ${\hat{\mu }}_{i}$ is obtained in Step 1, ${\left({\hat{\Sigma }}_{Y_{i}}\right)}_{j,l}=\hat{G}\left(T_{ij},T_{il}\right)+{\hat{\sigma }}^{2}\delta_{jl}$ is estimated in Step 2, and ${\hat{\phi }}_{ik}=\left(\phi_{k}\left(T_{i1}\right),...,\phi_{k}\left(T_{iN_{i}}\right)\right)$ and ${\hat{\lambda }}_{k}$ is obtained from Step 3.
  For our data analysis, we use the MATLAB package, Principal Analysis by Conditional Expectation (PACE) [17], for the implementation of FPCA.

Typically, the first few PCs explain the largest fraction of total variation in all the trajectories, thus represent the dominant modes of variation. For each individual, the trajectory can be well approximated by a linear combination of these first *K* smooth functions. Popular approaches to choose *K* include the Akaike information criterion (AIC) and fraction of variance explained (FVE). We employ the FVE method with a threshold of 0.99 of the variance explained. That is, we choose the smallest *K* eigenfunctions that explain at least 99% of the total variance. Once *K* is chosen we approximate the trajectory of the *i*-th subject as

$$\begin{array}{c} {\hat{X}}_{i}^{K}\left(t\right)=\hat{\mu }\left(t\right)+\sum_{k=1}^{K}{\hat{\xi }}_{ik}{\hat{\phi }}_{k}\left(t\right), \end{array}$$ (5)

where ${\hat{\xi }}_{ik}$’s are the individual-specific FPC scores defined in (4). In (5), the terms $\hat{\mu }\left(t\right)$ and ${\hat{\phi }}_{k}\left(t\right)$ borrows information from the entire data set, while ${\hat{\xi }}_{ik}$ is driven by ${\tilde{Y}}_{i}$, the observations of the specific subject. As described previously, we have up to 5 weight records for each subject in our study measured from *t* = 0 (*W*_0_) to *t* ≥ 37 (*W*_*H*_ at GA at birth). Individual weight trajectories can be estimated following steps 1 to 4 outlined above. Simultaneous confidence interval bands for individual trajectories are also obtained; details on the construction of confidence interval bands are given in Appendix A.

#### Performance comparison: FPCA vs. non-linear mixed effect model for trajectory modeling

**Fig 1:**
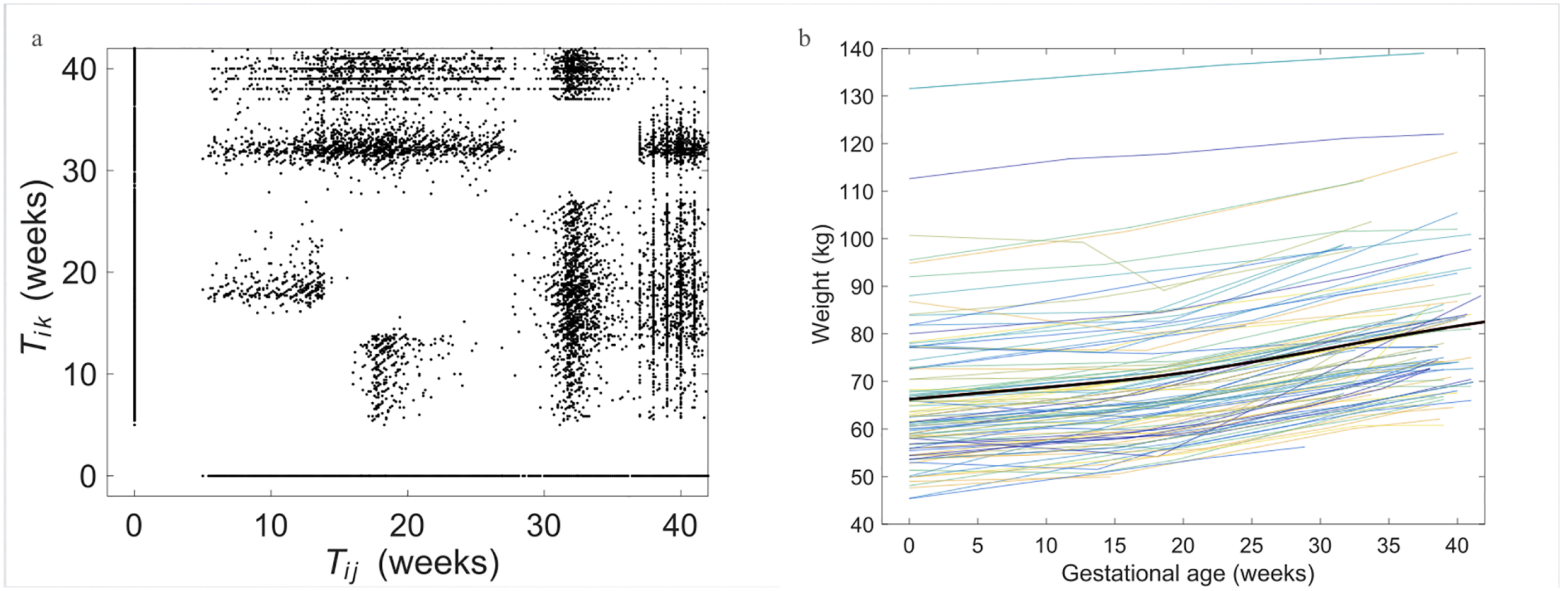
(a) Assembled pairs (*T*_*ij*_, *T*_*ik*_) of all weight records. (b) Individual weight trajectories of 100 randomly selected subjects, overlaid with the smooth estimate of the mean function.

The performance of the FPCA-based individual trajectory is compared with the classic model for trajectory modelling. Since the weight growth curve is nonlinear (Fig 1), we choose a parametric nonlinear mixed effect model. It is also observed that the mean weight curve is convex during early and mid-pregnancy and there is an inflection point at 30 weeks (Fig 1). Thus we use a logistic curve to model this shape of weight trajectory, which is in the form of

$$\begin{array}{c} W\left(t\right)=\frac{L}{1+\exp(-k\left(t-t_{0}\right))}. \end{array}$$

As a logistic curve always starts from zero, we add an intercept *c* to this model. The model then has the following form:

$$\begin{array}{c} W\left(t\right)=\frac{L}{1+\exp(-k\left(t-t_{0}\right))}+c. \end{array}$$ (6)

In (6), *L* is the maximum of of the curve, which is related to the total weight gain. Since we use only part of the curve, it acts as a magnifying factor. *k* is the steepness of the curve, which is affected by the weight growth rate. *t*_0_ is an inflection point. From the shape of the estimated mean function, we set it at 30 weeks. *c* is a baseline weight and can be interpreted as the prepregnancy weight. *L* and *c* are treated as random effects and *k* is treated as a fixed effect of the model to be estimated from the data.

After estimating the weight trajectory of each subject with models 5 and 6, we compare the model-based weight estimates at each study visit to the observed weights for each subject to quantify the model fit. The mean squared error (MSE) and root mean squared error (RMSE) is thus calculated as

$$\begin{array}{c} MSE=\frac{1}{\sum_{i=1}^{n}N_{i}}\sum_{i=1}^{n}\sum_{j=1}^{N_{i}}{\left(\hat{W}\left(T_{ij}\right)-W\left(T_{ij}\right)\right)}^{2},\;RMSE=\sqrt{MSE} \end{array}$$

#### Gestational weight change

The gestational weight change can be defined either absolutely or relatively. The conventional gestational weight change is defined by total GWG, which is the weight at delivery minus the prepregnancy weight. As weight at delivery is unavailable in our data set, it is substituted with *W*_*C*_ or *W*_*H*_ whichever is larger. Thus the absolute change in weight is:

$$\begin{array}{c} G=\max\left\{{\text{W}}_{H},{\text{W}}_{\text{C}}\right\}-{\text{W}}_{0}. \end{array}$$ (7)

This GWG has three potential drawbacks: (i) For subjects whose self-reported *W*_*H*_ is less than measured *W*_*C*_, the latter is used as the highest weight. However, we know from our data that subjects are unlikely to schedule a visit after 37 weeks of GA. *W*_*C*_ measured before 37 weeks of GA is likely differ from weight at delivery; (ii) It is possible that the weight of a pregnant woman drops during the last few weeks of gestation. The highest weight may differ from her weight at delivery; (iii) Both *W*_*H*_ and *W*_0_ are self-reported and subject to bias in recall and reporting [28, 29]. Thus, absolute weight change as defined in (7) may overestimate the total GWG. Alternatively, estimated total GWG can be obtained from the weight trajectory given by FPCA, denoted by G’:

$$\begin{array}{c} G^{\prime }=\left(\text{weight}\;\text{estimated}\;\text{at}\;\text{GA}\;\text{at}\;\text{delivery}\right)-\left(\text{weight}\;\text{estimated}\;\text{at}\;\text{GA}=0\right). \end{array}$$

The relative weight change is expressed as a ratio of weight at delivery and pre-pregnancy weight. A log-transformed weight ratio is denoted by LG and LG’, respectively:

$$\begin{array}{c} LG=\log\left(\max\left\{W_{H},W_{C}\right\}\right)-\log\left(W_{0}\right)=\log\left(\frac{\max\{W_{H},W_{C}\}}{W_{0}}\right), \end{array}$$

$$\begin{array}{c} LG^{\prime }=\log\frac{\text{weight}\;\text{estimated}\;\text{at}\;\text{GA}\;\text{at}\;\text{delivery}}{\text{weight}\;\text{estimated}\;\text{at}\;\text{GA}=0}. \end{array}$$

*G*, *G*′, *LG* and *LG*′ are the four possible response variables for the regression model. The covariates include prepregnancy BMI, nutrient intakes, and PA. In the APrON study, nutrient intakes and PA indices were collected longitudinally during the same study visit when the weight was measured.

To fit a multiple linear regression model, we use the average of all relevant measurements from one subject as her covariate value for the nutrient intake and PA.

We have also directly modelled the estimated weight trajectory as the response variable and the estimated trajectories of nutrient intakes and PA as the explanatory variables, extending the approach by Müller et al. [30]. The results from this approach are not reported because the complex model with trajectory as covariates has identical *R*^2^ and MSE as the much simpler and more straightforward multiple linear regression model.

## Results

The APrON study recruited 2191 pregnant women, among which 1629 subjects have full-term, singleton, live births, and at least one weight record. 101 were excluded because they completed neither food recall nor Baecke’s PA questionnaire. We further excluded 6 subjects who completed both the interviewer-administered and web-based versions of food recall. In our final cohort, 725 completed only the interviewer-administered version of the 24-hour food recall, 797 completed only the web-based version of the 24-hour food recall.

### Modeling the trajectories of weight with FPCA

The distribution of the time points when the observations were made is revealed by the assembled pairs of time (*T*_*ij*_, *T*_*ik*_), where *i* indexes subject and *j*, *k* index time points. The assembled pairs of all the weight records of the 1540 subjects are shown in Fig 1(a). Note that by assigning *W*_*H*_ to GA at delivery, a larger cluster occurs at *T*_*ij*_ ≥ 37. The self-reported prepregnancy weight is assigned to GA = 0, resulting in the horizontal and vertical lines *T*_*i*1_ = 0. Both lines approximately start at 5 and ends at 42 weeks, indicating that there exists a weight record from a certain subject at almost any time point after 5 weeks. A smoothed mean weight trajectory is estimated for all subjects using the FPCA method (Fig 1(b)).

**Fig 2:**
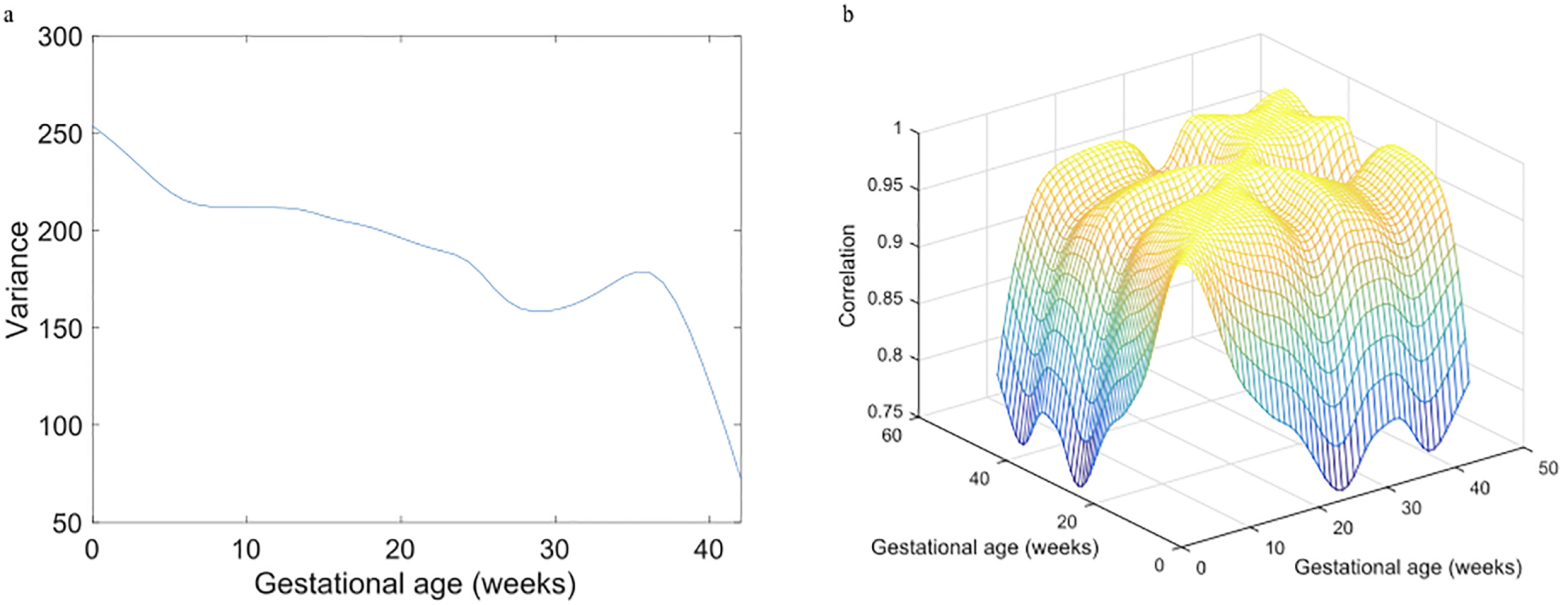
(a) Smooth estimate of the variance function of the weight data; (b) Smooth estimate of the correlation surface.

The smooth estimate of the variance function for weight data is shown in Fig 2(a). It decreases prior to 25 weeks of gestation, suggesting that variation in the weights are largest at the beginning of pregnancy. As the measurement times were schedule by trimesters, there are few measurement at the boundary between two trimesters. Specifically, there are only a few data points around GA = 29 (Fig 1), the estimate of variance around *t* = 29 is not as reliable as estimates elsewhere where more data points were available. Thus, the fluctuation observed around 27-35 weeks of gestation (Fig 2(a)) is more likely due to a lack of data than due to a true underlying pattern. After 35 weeks of gestation, the variance decreases rapidly, which indicates a smaller variance among *W*_*H*_ than *W*_0_ of all subjects.

The smooth estimate of the correlation surface of weight data is shown in Fig 2(b). The entire surface is above 0.7, indicating that weight records of the same subject are highly correlated at all times. However, the correlation between the weight during the first 20 weeks of gestation and weight measured at later times decreases drastically. Correlation between *W*_0_ and the gestational weight is weaker in later pregnancy (>20 weeks) than that in early pregnancy. It suggests that a subject’s weight begins to develop new patterns after 20 weeks. A strong correlation of weight within second/third trimester indicates that the weight at any two time points after a woman enters second trimester are highly correlated.

**Fig 3:**
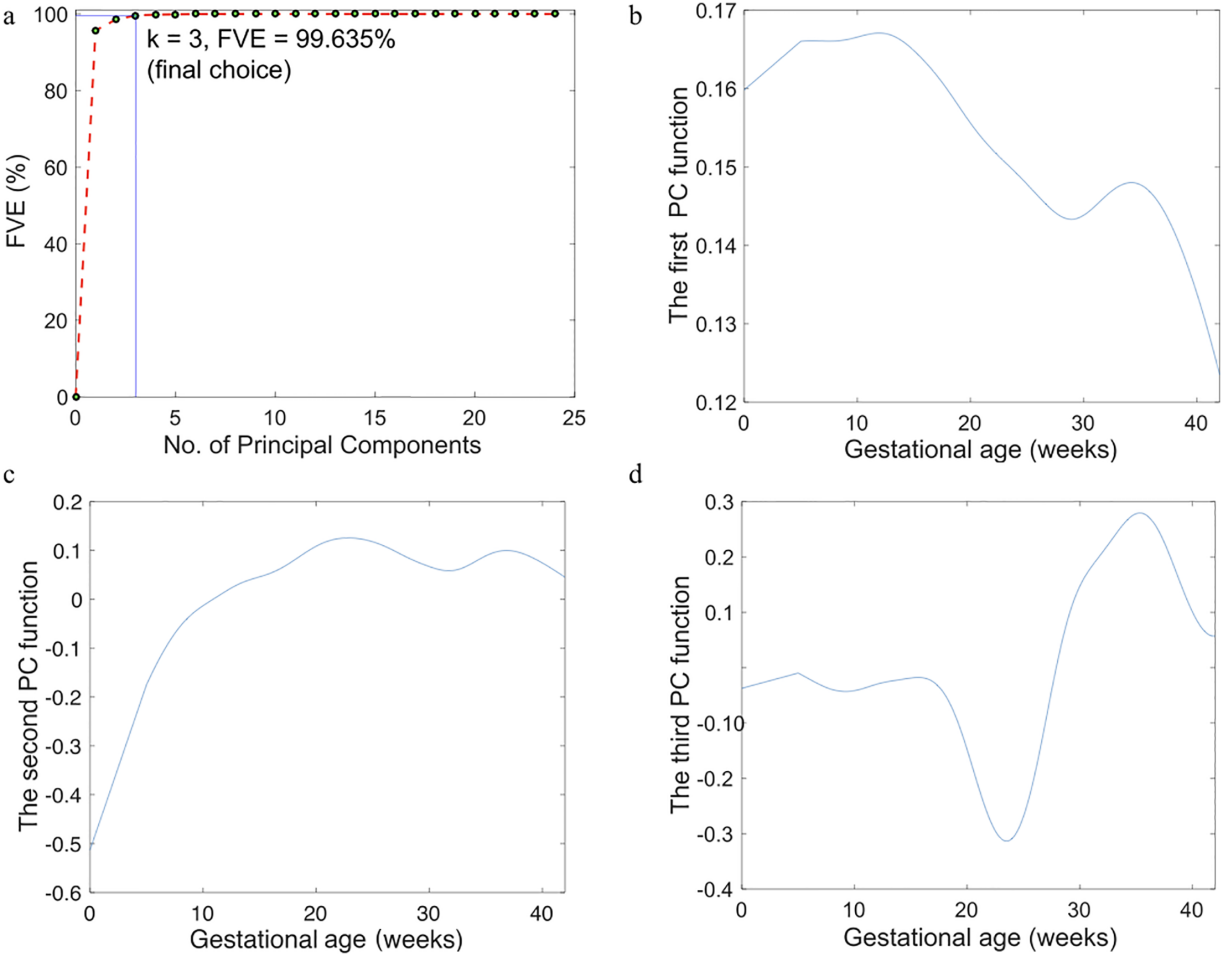
(a) Scree plot of the weight data and (b–d) The first, second and third principle component (PC) functions for the weight data which account for 95.7%, 2.8%, and 1.1% of the total variation, respectively.

The scree plot is shown in Fig 3(a). The first 3 PCs account for more than 99% of the variation, thus they are used to estimate an individual’s weight trajectory. Accounting for 95.7% of the total variation, the first PC is an average over the pregnancy, which is flat during the first trimester, and decreases rapidly in the second and third trimester (Fig 3(b)). It corroborates the fact that the majority of weight is gained during the last two trimesters. The second PC increases most rapidly in the first trimester, corresponding to a contrast between weights in pre- or very early pregnancy and the second/third trimester (Fig 3(c)). The third PC (Fig 3(d)) corresponds to a contrast between the second and the third trimester.

**Fig 4:**
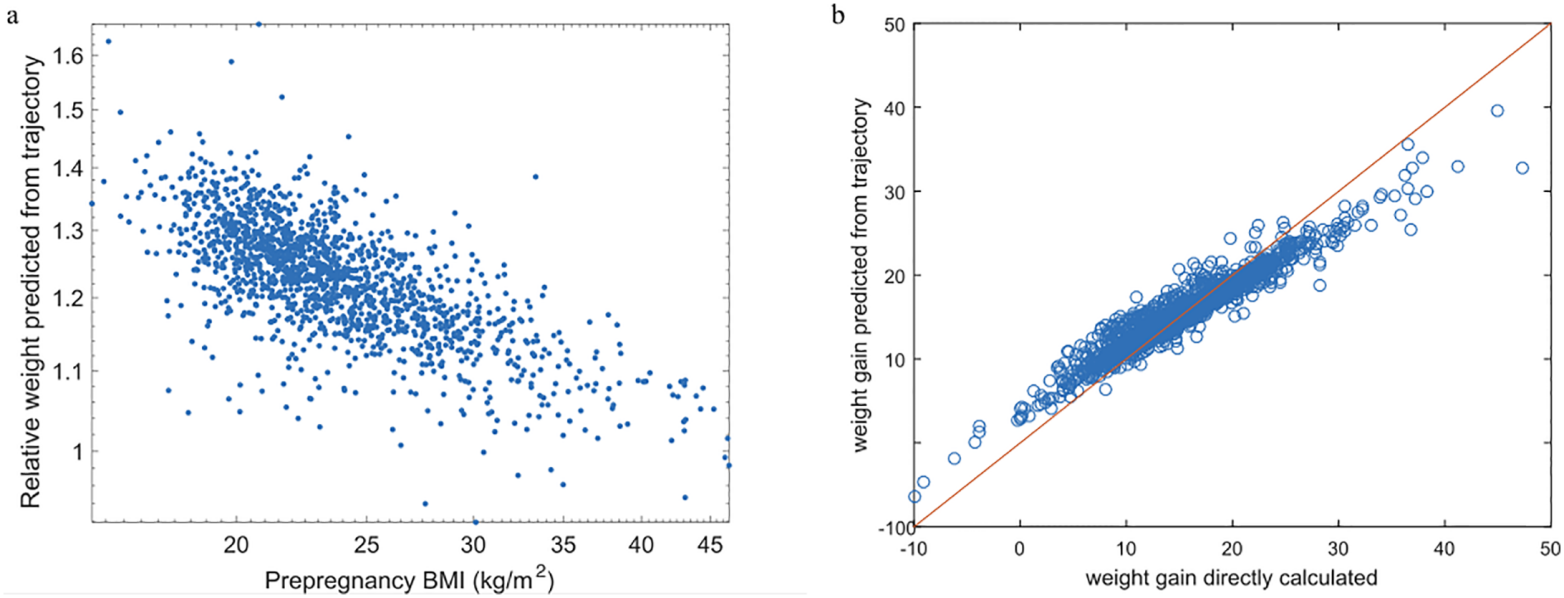
(a) Scatter plot of the estimated relative weight (*W*_*d*_/*W*_0_) from the trajectory on log-scale vs. prepregnancy BMI on log-scale (*W*_*d*_: weight at birth; *W*_0_: prepregnancy weight) and (b) Weight gain predicted from the trajectory vs. weight gain directly calculated.

The weight gain estimated from trajectory could account for the weight gain in the last few weeks even if the subject does not have any weight record. Moreover, FPCA method has a smoothing effect. For subjects with large/small weight gain, the estimated trajectory tends to have a lowered/increased weight gain, and thus the estimated weight gain is closer to the mean weight gain. This effect can be seen from Fig 4(b). The weight gain estimated from the trajectory, *G*′, is highly correlated with the weight gain directly calculated, *G*, but tends to be smaller than *G* when *G* is large, or larger than *G* when *G* is small. This approach of estimating GWG may reduce the bias of under-reporting the prepregnancy BMI or over-reporting the *W*_H_, as noted in [28, 29].

### Comparison of FPCA and non-linear mixed effect model for weight trajectory

**Fig 5:**
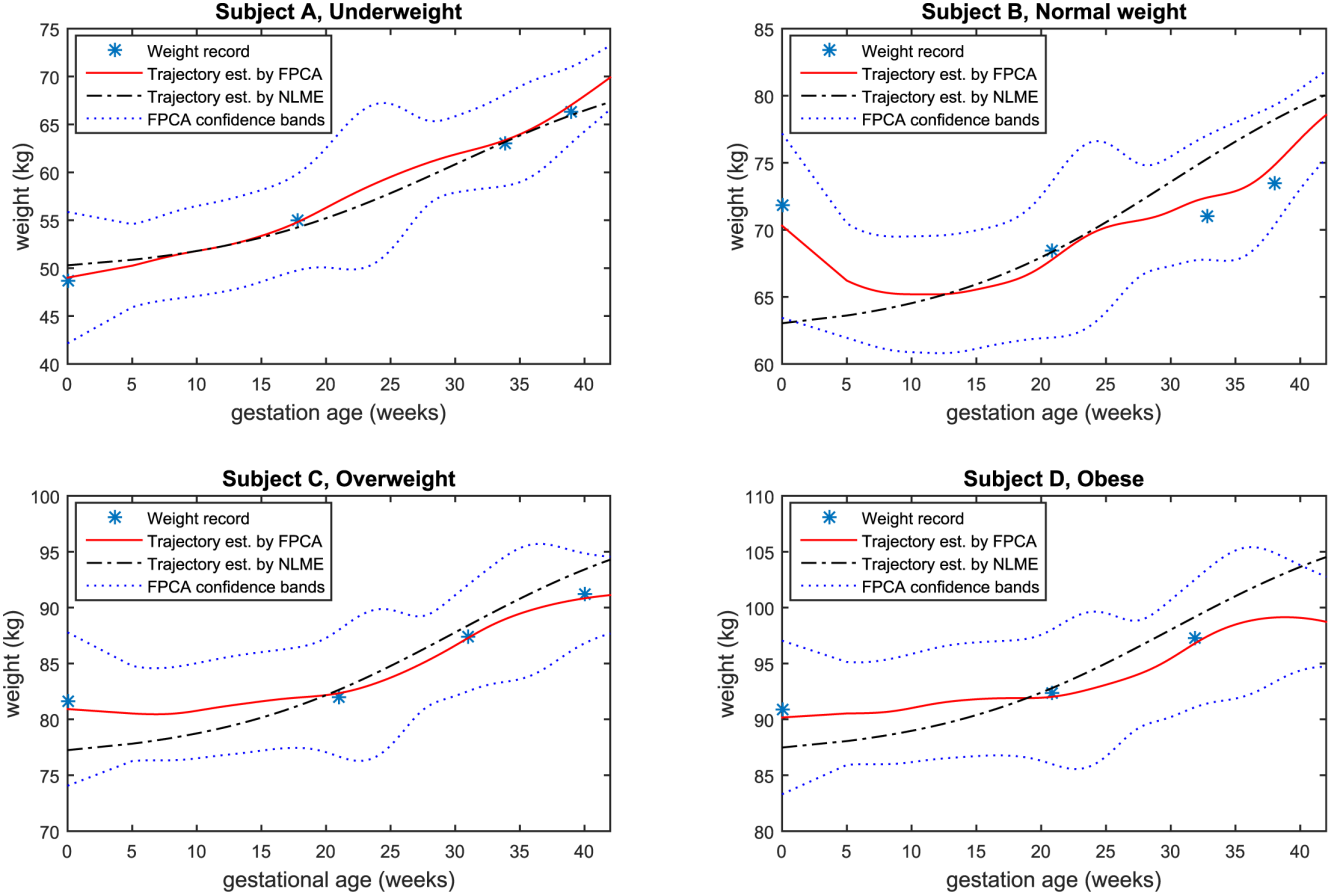
Estimated weight trajectories of four subjects, one from each prepregnancy weight categories.

Fig 5 displays observed weights during pregnancy from four study subjects, one from each prepregnancy BMI category as defined by the Institute of Medicine [5]. The estimated trajectories using the FPCA model (5) and NLME model estimated in the form of (6) are superimposed in the same panel. For subject A, her weight steadily increases during pregnancy, which is of similar pattern to the mean function of all samples; see Fig 1. Estimated trajectories from both FPCA and NLME are in good agreement with the observed weights. Subject B lost weight at the beginning of her pregnancy and did not regain to her prepregnancy weight even in the 3rd trimester. So her weight gain pattern deviates from the mean function of all samples. The NLME approach uses a curve shape similar to subject A (and in fact, similar to the mean function of all samples), so the fitted trajectory gives poor estimates for her observed weights that yields large residuals $|\hat{W}\left(T_{ij}\right)-W\left(T_{ij}\right)|$. The trajectory estimated by FPCA gives much better estimates for subject B’s observed weight.

As illustrated in Fig 5, FPCA provides a flexible fit to the weight data and a significantly better fit to the weight trajectory than the classic NLME model. The superior model fit is also confirmed by a 35% reduction in residual variance when FPCA is compared to NMLE (4.4kg^2^ FPCA vs. 6.8kg^2^ NLME).

We also applied FPCA model to the sparse longitudinal nutrient intake data and PA indices to estimate their trajectories. However, these attempts did not yield satisfactory fit (results not shown).

### Explaining variation in gestational weight change

We successfully estimated the trajectories of weight from sparse measurements, which may be used to predict the total GWG and inform interventions such as counseling and education. Our next goal is to identify the factors associated with the weight change during pregnancy, such as prepregnancy BMI, caloric intakes and physical activity.

Since we observed a nonnegligible discrepancy in caloric intakes calculated from the two 24-hour food recall instruments [24], we built separate models, stratifying participants by food recall instrument. The results from the two separate models are similar. Thus, we presented the results from modeling participants who completed the interview-administrated 24h food recall as an example to illustrate our findings. Our explanatory variables are prepregnancy BMI, the average total caloric intake and the average of the PA indices. Table 1 reports the RMSE and *R*^2^ of these regression models.

**Table 1:** Comparison of the regression models with different forms of responses and predictors.

Response	*R* ^2^(RMSE)	
	BMI_*p*_	BMI_*p*_, $\bar{T}$, $\bar{TI}$
*G*	0.05 (6.25)	0.08 (6.09)
*LG*	0.27 (0.07)	0.29 (0.07)
*G*′	0.22 (3.91)	0.25 (3.81)
*LG*′	0.51 (0.05)	0.52 (0.05)

BMI_*p*_: prepregnancy BMI (kg/m^2^); $\bar{T}$: Average total calorie intakes during pregnancy; $\bar{TI}$: Average physical indices during pregnancy. All the models are using the data captured by the interviewer-administered instrument.

We observe the following: 1) The weight change as estimated from the weight trajectory can be explained better than the weight change calculated from the weight records; 2) The relative weight change (log-transformed) can be explained better than the absolute weight change; 3) Adding nutrient intake and PA data did not improve model fit as measured by the RMSE or *R*^2^, when compared to the corresponding simple linear regression model with prepregnancy BMI (BMI_*p*_) as the only predictor. Among these models, the most parsimonious model is given by

$$\begin{array}{c} LG^{\prime }=\beta_{0}+\beta_{1}{\text{BMI}}_{p}, \end{array}$$

where the response variable is the log transformed relative weight change calculated from the estimated weight trajectory and the explanatory variable is the prepregnancy BMI, which explains 51% of the variation in weight changes during pregnancy. Fig 4(a) shows a scatter plot of the relative weight change and prepregnancy BMI (both on log-scale), illustrating a strong negative linear association between prepregnancy BMI and weight change during pregnancy.

## Discussion

Meeting GWG recommended by guideline was achieved only in a minority of the pregnancies [7]. A useful intervention is to combine customized counseling and education with individual weight monitoring [9, 10], which could benefit from a personalized weight growth trajectory as a reference for targeted intervention. Our study demonstrated that the FPCA approach successfully captures the shape of the individual gestational weight growth trajectory with sparse (3 to 5 observations per subject) irregularly-spaced gestation weight (Fig 1). FPCA has the potential to be developed into a tool for predicting gestational weight growth. In addition, we found that the variation in gestational weight change estimated from these individualized trajectories can be largely explained by woman’s prepregnancy BMI. Contrary to our expectation, adding the dietary intake and physical activity covariates, either as trajectories or numeric averages, does not improve the explained variation of weight changes. The most likely explanation is that dietary intake and physical activity data are subject to large variation (e.g. day-to-day variation in diet and physical activities), as well as large measurement errors due to the nature of survey data. Moreover, the dietary intake and physical activity data are more sparse than the weight data, making accurate trajectory modeling more challenging.

When modeling the individual weight trajectory with our sparse, irregularly collected longitudinal data, FPCA offers a significant advantage when compared to the NLME, a classic approach in modeling individual curves [14–16]. As seen in Fig 5, the curves estimated using NLME all follow a fixed shape of the mean weight trajectory of the population. This is because once the function is specified, the estimated trajectories can only differ by a dilation and shift but maintain the shape of the curve. Thus, NLME can not capture large deviation in individual patterns from the mean weight trajectory. It is also known that when there are only three or four data points per subject, the parameters of NLME estimated using either restricted maximum likelihood or maximum likelihood are prone to be biased. In contrast, the curve estimated using FPCA is flexible and adaptive to the observed sparse data points, which is especially useful for individuals who have different weight trajectories from the mean weight trajectory of the population. For each subject we have only up to five weight records. In the four steps of our estimation, estimation of both the PFC scores and eigenfunctions relies on the estimate of the covariance surface. As long as the total covariance structure is consistently estimated, which relies on sufficient density of the assembled pairs (*T*_*ij*_, *T*_*ik*_), the estimate of trajectory works reasonably well.

The principle components identified by the FPCA offers insight into the weight trajectories among subjects. The first principle component is flat in the first trimester then rapid declines, which corresponds to the overall pattern of bodyweight variances during pregnancy. That is, as gestational age increases, the variation in body weight decreases. This trend agrees with our second study finding that the estimated total GWG is negatively correlated with prepregnancy BMI and more than half of the variation in total GWG are explained by prepregnancy BMI alone. This strong negative correlation between GWG and prepregnancy BMI is consistent with the GWG guidelines from IOM [31], which recommends women in a lower prepregnancy BMI category to gain more weight during pregnancy than those in a higher prepregnancy BMI category.

Within subject correlation provides insight into individual patterns of GWG. The correlation between the weights at the beginning and towards the end of the pregnancy was low. It suggests that in general, women’s weight develops a new pattern after 20 weeks into gestation. This within subject weight correlation pattern suggests that an efficient time to start predicting weight growth is likely to be in the second trimester for the majority of pregnant women.

The FPCA method borrows information from the entire data set, and does not incorporate women’s prepregnancy BMI information. However, women with lower prepregnancy BMIs tend to have higher GWG and their weight growth trajectories are likely steeper than the mean weight growth trajectory. Women with higher prepregnancy BMIs have an opposite trend; their weight trajectories are more gradually rising than the mean weight growth trajectory. For our future research, we will address this limitation and improve fit by incorporating additional BMI category-specific FPCAs.

## Appendix A: Confidence bands of individual trajectories

Let *K* be the number chosen using FVE approach, and ***ξ***_*K*, *i*_ = (*ξ*_*i*1_, …, *ξ*_*iK*_)^*T*^, ${\tilde{\xi }}_{K,i}$ $={\left({\tilde{\xi }}_{i1},...,{\tilde{\xi }}_{iK}\right)}^{T}$ be the vector of FPC scores. The covariance matrix of ${\tilde{\xi }}_{K,i}$ can be written as $H\Sigma_{Y_{i}}^{-1}H^{T}$, where *H* is the covariance matrix between ***ξ***_*K*,*i*_ and ${\tilde{Y}}_{i}$. Note that for a fixed sample, *λ*_*k*_, *ϕ*_*ik*_ and ${\left({\hat{\Sigma }}_{Y_{i}}\right)}_{j,l}=\hat{G}\left(T_{ij},T_{il}\right)+{\hat{\sigma }}^{2}\delta_{jl}$ are independent with ${\tilde{Y}}_{i}$, so ${\tilde{\xi }}_{K,i}$ is a linear function of ${\tilde{Y}}_{i}$. *H* can be rewritten as

$$\begin{array}{c} H={\left(\lambda_{1}\phi_{i1},\cdots ,\lambda_{K}\phi_{iK}\right)}^{T}. \end{array}$$ (1)

The estimation error of ${\tilde{\xi }}_{K,i}$ can be assessed by $\text{var}({\tilde{\xi }}_{K,i}-\xi_{K,i})$. The conditional expectation $E(\xi_{K,i}|{\tilde{Y}}_{i})$ is the projection of *ξ*_*K*,*i*_ on the space $\text{span}\{{\tilde{Y}}_{i}\}$, thus $E\left({\tilde{\xi }}_{K,i}\xi_{K,i}^{T}\right)=E\left({\tilde{\xi }}_{K,i}{\tilde{\xi }}_{K,i}^{T}\right)$, and

$$\begin{array}{c} \text{var}\left({\tilde{\xi }}_{K,i}-\xi_{K,i}\right)=\text{var}\left(\xi_{K,i}\right)-\text{var}\left({\tilde{\xi }}_{K,i}\right)=\Omega_{K}, \end{array}$$

where $\Omega_{K}=\Lambda -H\Sigma_{Y_{i}}^{-1}H^{T}=\text{diag}\left(\lambda_{1},...,\lambda_{K}\right)-H\Sigma_{Y_{i}}^{-1}H^{T}$. Under Gaussian assumptions, we have $\left({\tilde{\xi }}_{K,i}-\xi_{K,i}\right)∼N\left(0,\Omega_{K}\right)$. From (5), the individual trajectory is estimated as ${\hat{X}}_{i}^{K}\left(t\right)=\hat{\mu }\left(t\right)+\sum_{k=1}^{K}{\hat{\xi }}_{ik}{\hat{\phi }}_{k}\left(t\right)$. It was showed that ${\hat{X}}_{i}^{K}\left(t\right)-X\left(t\right)$ approximately follows normal distribution $N(0,{\hat{\phi }}_{K,t}^{T}{\hat{\Omega }}_{K}{\hat{\phi }}_{K,t})$, where ${\hat{\phi }}_{K,t}={\left({\hat{\phi }}_{1}\left(t\right),...,{\hat{\phi }}_{K}\left(t\right)\right)}^{T}$, ${\hat{\Omega }}_{K}=\hat{\Lambda }-\hat{H}{\hat{\Sigma }}_{Y_{i}}^{-1}{\hat{H}}^{T},$ with $\hat{\Lambda }=\text{diag}\left({\hat{\lambda }}_{1},...,{\hat{\lambda }}_{K}\right)$, $\hat{H}={\left({\hat{\lambda }}_{1}{\hat{\phi }}_{i1},\cdots ,{\hat{\lambda }}_{K}{\hat{\phi }}_{iK}\right)}^{T}$. Therefore, the (1 − *α*) asymptotic pointwise confidence intervals for individual trajectories are given as

$$\begin{array}{c} {\hat{X}}_{i}^{K}\left(t\right)\pm \Phi^{-1}\left(1-\alpha /2\right)\sqrt{{\hat{\phi }}_{K,t}^{T}{\hat{\Omega }}_{K}{\hat{\phi }}_{K,t}}. \end{array}$$ (2)

The (1 − *α*) asymptotic simultaneous confidence intervals for individual trajectories are given as

$$\begin{array}{c} {\hat{X}}_{i}^{K}\left(t\right)\pm \sqrt{\chi_{K,1-\alpha }^{2}{\hat{\phi }}_{K,t}^{T}{\hat{\Omega }}_{K}{\hat{\phi }}_{K,t}}. \end{array}$$ (3)

## Supporting information

S1 File Questions for participants on *W*_0_ and *W*_*H*_. (DOCX)

S2 File MATLAB and R codes for data analysis. (ZIP)
