# Supplementary material for: Trajectory modeling of gestational weight: A functional principal component analysis approach
Source: PLoS One. 2017 Oct 24;12(10):e0186761. doi: 10.1371/journal.pone.0186761 (PMC5655493; doi:10.1371/journal.pone.0186761)
Supplement: S1 File — (DOCX) [file pone.0186761.s001.docx]

Questions for participants on pre-pregnancy weight and highest weight during pregnancy

Pre-pregnancy weight was collected at the first study visit, determined from the following question:

**“How much did you weigh immediately before you were pregnant?”**

Highest weight in pregnancy was collected at the study visit that took place at approximately 3 months postpartum, determined from the following question:

**“What was your highest weight in pregnancy?”**
